# From Corncob By-Product to Functional Lignins: Comparative Analysis of Alkaline and Organosolv Extraction Followed by Laccase Treatment

**DOI:** 10.3390/biom15091226

**Published:** 2025-08-26

**Authors:** Elise Martin, Swarnima Agnihotri, Fabrice Audonnet, Eric Record, Pascal Dubessay, Mohammad J. Taherzadeh, Philippe Michaud

**Affiliations:** 1 Clermont Auvergne INP, CNRS, Institut Pascal, Université Clermont Auvergne, 63000 Clermont-Ferrand, France; elise.martin@uca.fr (E.M.); pascal.dubessay@uca.fr (P.D.); philippe.michaud@uca.fr (P.M.); 2 Swedish Centre for Resource Recovery, Faculty of Textiles, Engineering and Business, University of Borås, 503 32 Borås, Sweden; swarnima.agnihotri@hb.se (S.A.); mohammad.taherzadeh@hb.se (M.J.T.); 3 INRAE, Aix-Marseille Université, UMR1163 Biodiversité et Biotechnologie Fongiques, 13288 Marseille, France; eric.record@inrae.fr

**Keywords:** lignin, extraction, corncob, laccases, *Pycnoporus cinnabarinus*

## Abstract

Corncobs, produced globally at over 200 million tons annually with 11–18% lignin content, represent an abundant and underexploited lignocellulosic resource for sustainable lignin valorization. In this study, two distinct extraction methodologies, alkaline treatment using sodium hydroxide and an organosolv process with a 50:50 ethanol/water mixture, were systematically compared for their efficiency in isolating lignin from corncobs. Both protocols achieved high yields, up to 82% for alkaline and 84% for organosolv extraction under optimized conditions. The resulting lignins displayed notable differences in chemical structure and physical properties, as revealed by spectroscopic and thermal analyses, highlighting their divergent potential for downstream applications. To evaluate the suitability of these lignins to biocatalytic upgrading, post-extraction enzymatic treatment was performed using *Pycnoporus cinnabarinus* laccase (EC 1.10.3.2). Significant structural modifications were observed in alkaline-extracted lignin, as determined by FTIR spectroscopy, while organosolv lignin remained largely unaltered, a difference attributed to its lower aqueous solubility at the enzyme’s optimal pH. These results demonstrate the critical impact of extraction conditions on lignin reactivity and suitability for enzymatic tailoring. This work underscores the potential for holistic corncob valorization within integrated biorefinery frameworks. Selective extraction and targeted enzymatic modification not only facilitate efficient by-product utilization but also expand the prospects for producing versatile bio-based materials, thereby advancing the transition toward a sustainable, circular bioeconomy.

## 1. Introduction

Within the spectrum of forestry-derived products, lignin has emerged as a central topic. As a complex aromatic biopolymer found in the pectocellulosic matrix of vascular plant cell walls, it confers mechanical rigidity and acts as a protective chemical barrier against microbial degradation. Constituting one of the main components of lignocellulosic biomass, lignin is abundantly available as a by-product of the pulp and paper industries. However, it remains underexploited, primarily relegated to “low-value” applications, despite its potential for high value-ones. To cite a few examples, lignin can be used to produce aromatic molecules and other chemical products in a greener way [1], to replace phenol formaldehyde in some adhesive formulation [2], to design green hydrogels with biomedical, biotechnological, or tissue engineering applications [3], or be employed as adsorbents for treating contaminated wastewaters [4]. Other possible applications include food packaging, agriculture, or uses as antimicrobial agent [5]. The wide range of potential applications makes lignin extraction economically attractive and, importantly, offers a promising way to valorize it. In this context, laccases (EC 1.10.3.2) could be employed to develop advanced lignin-based biosourced materials, for example by grafting other molecules on lignin polymer or by catalyzing polymerization and depolymerization of lignin [6].

Corn is one of the most extensively cultivated and consumed cereal crops worldwide [7]. Global corn grain production has increased by approximately 40%, currently exceeding 1.2 billion tons annually [8]. Among these, corncobs, the hard core of an ear of maize, constitute a significant inedible by-product, among the three most prevalent agricultural residues worldwide and the most prevalent one in the United States [9]. On average, the production of 100 kg of corn grain results in approximately 18 kg of corncobs [10], underscoring their potential for valorization. The corncob typically contains 35–46 wt% cellulose, 28–42 wt% hemicellulose, and 11–18 wt% lignin [11].

This study focusses on the extraction of lignins from corncobs collected in the Auvergne Rhône-Alpes region, followed by their enzymatic modification using laccase-catalyzed reactions. Two extraction methods were employed: alkaline treatment using NaOH and an organosolv process. The rationale behind using two distinct extraction methods lies in the fact that the physicochemical properties of lignin, such as purity, hydrophobicity, molecular weight, and water solubility, vary significantly depending on the extraction technique [12,13]. These properties are especially important for laccase modifications occurring in aqueous media. Lignins with lower hydrophobicity are more amenable to enzymatic modifications, including depolymerization, repolymerization, and functionalization. The organosolv process typically yields lignins of higher purity and lower molecular weight. These lignins are hydrophobic, limiting their reactivity with laccases. Conversely, alkaline extraction, while generally less efficient, produces lignins that are more hydrophilic and therefore more compatible with aqueous enzymatic systems. For these reasons, both processes were evaluated and compared. Alkaline pretreatment is a widely used method for processing lignocellulosic biomass, employing bases such as NaOH, NH_4_OH, or Ca(OH)_2_. It is often favored due to its operational simplicity and its relatively mild reaction conditions, typically involving 0.5–10% NaOH at temperatures ranging from room temperature to 180 °C for 5–60 min (biomass loading of 10–30% *w*/*v*). This method is particularly effective for herbaceous biomass like corncobs, facilitating lignin and hemicellulose solubilizations and de-esterification of crosslinking bonds [14].

The organosolv process employs organic solvents, commonly alcohols (e.g., methanol, ethanol, and butanol), cyclic ethers (e.g., THF; dioxane), polyols (e.g., ethylene glycol; glycerol), or ketones (e.g., acetone), in combination with water to extract lignin. Solvent-to-water ratios typically range from 40 to 80% (*w*/*w*), and reactions are conducted at elevated temperatures (140–220 °C), often under pressure. The process efficiency can be further enhanced by the addition of acid catalysts such as HCl, H_2_SO_4_, or H_3_PO_4_ [15]. The organosolv process offers the advantage of producing high-purity, sulfur-free lignin with minimal structural degradation, making it more suitable for high-value applications compared to conventional extraction methods.

The primary objective of this study was to compare the efficiency of lignin extraction from corncobs using the alkaline and organosolv methods, and to characterize the structural features of the resulting lignins. These lignins were then subjected to enzymatic modification using fungal laccases, with a focus on understanding how their extraction-dependent properties influence their reactivity and potential for downstream applications.

## 2. Materials and Methods

### 2.1. Chemicals and Raw Material

Sodium hydroxide (NaOH) in pellets was purchased from Sigma-Aldrich (38070 Saint-Quentin-Fallavier, France).

A single batch of dried corncob (CC) of the corned-dent variety, harvested by Limagrain in the Limagne plain (45°57′33″ N, 3°35′38″ E, Ennezat, France), was used during this study. Corncobs were previously dried in the open air in silos and were collected and then stored at room temperature. The corncob is composed of ash (1.4–2 wt%), Klason lignin (11.2 wt%), and carbon (44 wt%). The corncob was ground and then sieved to a particle size of 1000–1600 µm.

### 2.2. Total Solids Determination

Total solids (TS) determination was performed by simply weighing a piece of the material whose total solids content is to be determined on a precision scale in a dry container of known weight as fully described in Technical Report NREL/TP-510-42621 [16]. The sample is then placed in an oven at 105 °C overnight to evaporate the water. The residue is then weighted again. The weight loss is used to calculate the TS content. In this study it will be expressed as a percentage.

### 2.3. Alkali Treatment

Ground corncob with a particle size between 1000 and 1600 µm was treated in NaOH solution (1–3% (*w*/*v*)) in a flask with a solid/liquid ratio of 1:20. Treatments were carried out for 1 or 24 h and temperature were ranging from 22 to 80 °C. The suspensions were stirred with a magnetic stirrer and heated if needed.

Different treatment conditions in duplicate were tested according to Table 1.

The remaining pulp was then filtered through a 0.45 mm mesh and washed with distilled water with two times the volume of reagent used. The washed pulp was air dried for 24 h. The filtrate was collected, and lignin was precipitated using 72% H_2_SO_4_ solution. Then, 72% H_2_SO_4_ solution was added to the filtrate until a pH of 3 is reached. The resulting solution was then centrifuged for 15 min at 5000 rpm (20 °C). The precipitate was collected and dried overnight at 105 °C before being ground to powder using a mortar and pestle.

### 2.4. Organosolv Process

The organosolv treatments were carried out on corncob in a 2 L stainless-steel benchtop Parr reactor (Parr Instrument Company, Moline, IL, USA) equipped with a stirrer, U-shaped cooling coil, heating jacket, and pressure and temperature sensor. A control unit (model 4848, Parr Instrument Company, Moline, IL, USA) was used to control the temperature and stirring. The effect of temperature on fractionation of the corncob by organosolv treatment was studied. The same retention time of 30 min and a solvent composed of 50% *v*/*v* ethanol were chosen for the experiments. For each condition, 30 g dry corncob and 570 g solvent were added to the reactor to have a total mass of 600 g. Prior to the addition to the reactor, the solvent pH was set at 3.5 using a 2 M H_2_SO_4_ solution.

After treatment, the reactor was cooled down, and the treatment slurry was emptied into a sieve with a pore size of 0.45 mm. The solid fraction was collected after washing with 300 mL of solvent at the same pH. The obtained liquid, known as black liquor, was diluted with 3 times its volume of water and then centrifuged at 5000× *g* for 10 min (Centrifuge 5804 R, Eppendorf, Hamburg, Germany) to separate the suspended solids.

Different treatment conditions were tested according to Table 2.

### 2.5. Laccase Assay

Laccase activity was measured by monitoring oxidation of 500 µmol·L^−1^ of 2,2′-Azino-bis-3-ethylbenzothiazoline-6-sulfonic acid (ABTS) at 420 nm (ε = 36,000 L·cm^−1^·mol^−1^) in an acetate buffer (100 mM, pH 4.5) at 25 °C for 1 min. The number of standard enzyme activity units (U) was expressed as μmol of ABTS oxidized per minute [17].

### 2.6. Enzymatic Modification of Lignins by Laccase

Alkali and organosolv lignins extracted from the corncob were suspended in Milli-Q water and adjusted to pH 4.5 with 1 M NaOH. Then, 100 mM acetate buffer pH 4.5 and Milli-Q water were added to obtain a final solution at 5% (*w*/*v*).

Lignins were treated using 50 U (per gram of lignin) of a high-redox-potential *Pycnoporus cinnabarinus* laccase (EC 1.10.3.2), produced and purified as previously described [17,18]. Enzymatic reaction was carried out at 25 °C for 2 h under shaking (350 rpm). Reactions were stopped by freeze-drying (−80 °C). Reactions were performed in triplicate.

### 2.7. Determination of Structural Carbohydrates and Lignin Biomass

Lignin and pulp samples were analyzed using the standard NREL method [19], fully described in Technical Report NREL/TP-510-42619. Each lignin or pulp sample is analyzed in duplicate. Three hundred mg of sample were weighed into a 100 mL Erlenmayer flask, and 3 mL of 72% H_2_SO_4_ were then added. A glass stir rod was used to mix the mixture in a 30 °C water bath for 60 min. The samples are stirred every 5 to 10 min without removing them from the bath. After 60 min, the acidic solution was diluted to 4% H_2_SO_4_ using deionized water. The samples were then incubated for 1 h at 121 °C. Once they were cooled down at room temperature, the hydrolysates were filtered through ashless Whatman filter papers with a 20–25 µm pore size. Acid-insoluble residues were dried at 105 °C and then placed in a muffle furnace at 575 °C for 24 h. The remaining residue was weighed to determine the ash content and the acid-insoluble residue. The filtrate containing acid-soluble residues was analyzed by measuring its absorbance at 320 nm.

### 2.8. Fourier-Transformed Infrared Spectroscopy (FTIR)

Lignin samples were analyzed in the 500–4000 cm^−1^ spectral region with a Thermo Scientific Nicolet iS5 FT-IR spectrophotometer (Thermo Fisher Scientific, Waltham, MA, USA) in attenuated total reflection mode (ATR diamond iD7), completing 32 scans at 4 cm^−1^ resolution. Data was collected and processed using OMNIC software (version 6.0).

### 2.9. Differential Scanning Calorimetry (DSC)

The glass-transition temperature (T_g_) of laccase-activated lignin was measured by both differential scanning calorimetry (DSC) and modulated differential scanning calorimetry (MDSC) following the same protocol as Malric-Garajova et al. [20] and using a TA Instruments Q2000 device (TA Instruments, New Castle, DE, USA). Data recording and analysis employed, respectively, TA Instrument Explorer and TA Universal Analysis software (TA Instruments, Guyancourt, France). Approximatively 7 mg of each lignin samples were sealed in an aluminum crucible. Analysis was performed under a nitrogen atmosphere (N_2_, 25 mL/min). The first phase, which aimed to erase the thermal history of samples, is a standard DSC heat/cool cycle with a ramp of 10 °C/min in a temperature range of 0–250 °C. The second phase, aimed to evaluate the T_g_ of lignin samples, was a MDSC heating ramp of 5 °C/min with an oscillation period of 60 s and an amplitude of ±0.796 °C in the same temperature range of 0–250 °C.

## 3. Results and Discussion

### 3.1. Alkali Treatment of Corncob

**Figure 1:**
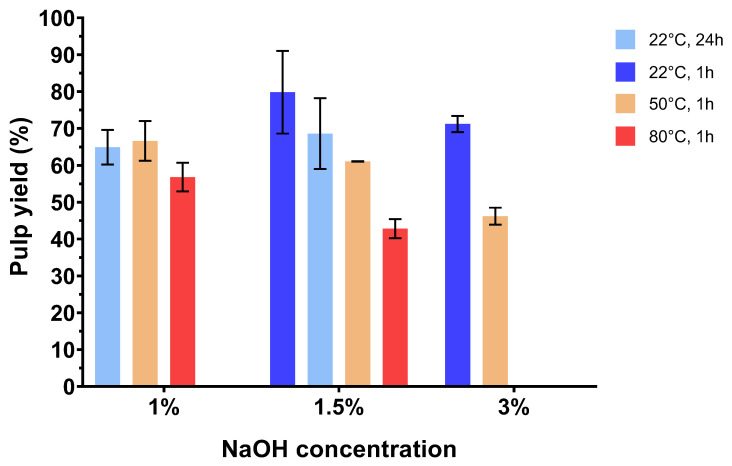
Pulp yields obtained from corncob after different alkali treatments.

**Figure 2:**
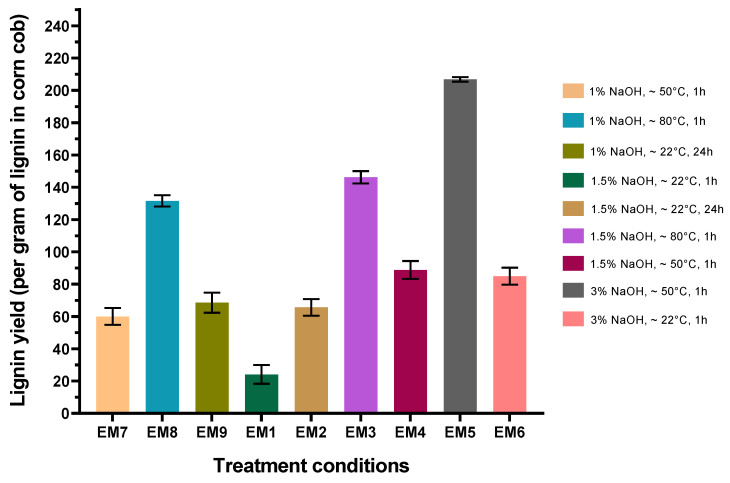
Lignin yields obtained from corncob after different alkali treatments.

Alkali treatment conditions were selected based on the previous unpublished work carried out at the University of Borås, which had shown that NaOH concentration, temperature, and treatment time were the most influential parameters. The weight loss of the pulp part (made of the glucan part of biomass), generally associated with lignin removal, was carefully followed, and the lignin yield obtained after acidic precipitation and drying was also calculated. All the results are summarized in Figure 1 and Figure 2.

Pulp yield, defined as the amount of solid fibrous material (pulp) remaining after pretreatment, relative to the original dry biomass, is a great indicator of the efficiency of alkaline extraction. Roy et al. [21] have explained that weight loss of fibers is caused by the gradual removal of hemicellulose and lignin. The alkali treatment breaks hydrogen bonds between the hydroxyl groups of the cellulose, hemicellulose, and lignin, leading to defibrillation and thus breakdown of the lignocellulosic material. As expected, in our case (Figure 1), at the same temperature and reaction time, for example 50 °C for 1 h, the increase in NaOH concentration led to a decrease in the pulp yield, from 67% for 1% NaOH to 61% for 1.5% to 46% for 3% NaOH. Similarly, at same NaOH concentration and reaction time, for example 1.5% NaOH and 1 h, an increase in the temperature leads to a decrease in the pulp yield after treatment, with 80% for 22 °C, 61% for 50 °C, and 43% for 80 °C. A longer reaction time leads to a non-significant decrease in yields. Thus, this last parameter seems to be the least influential of the three. The pulp yields obtained were correlated with those of lignins, as the more the fibers were broken down, the more lignin was recovered. In the same way, as observed for pulp yield, the conditions that led to the recovery of the most lignin were those involving higher NaOH concentrations and temperatures, with, for example, a yield of lignin (expressed per gram of lignin initially present in corncob) of 89% for 1.5% NaOH at 50 °C versus 24% for 1.5% NaOH at 22 °C (Figure 2). Similar temperatures and reaction times with different NaOH concentrations led to higher yield of lignin for higher concentrations in NaOH with 89% for 1.5% NaOH at 50 °C versus 208% for 3% NaOH at 50 °C.

Several NaOH concentrations showed lignin yields superior to 100% as, for too harsh processing conditions, fibers were completely denatured and lignin was probably polluted by hemicellulose and C6 and C5 sugars, which also precipitates at an acidic pH.

**Figure 3:**
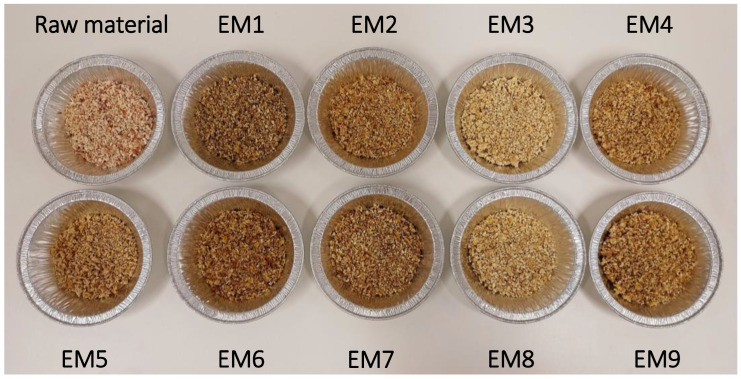
Picture of pulp samples recovered after different alkali treatments.

**Figure 4:**
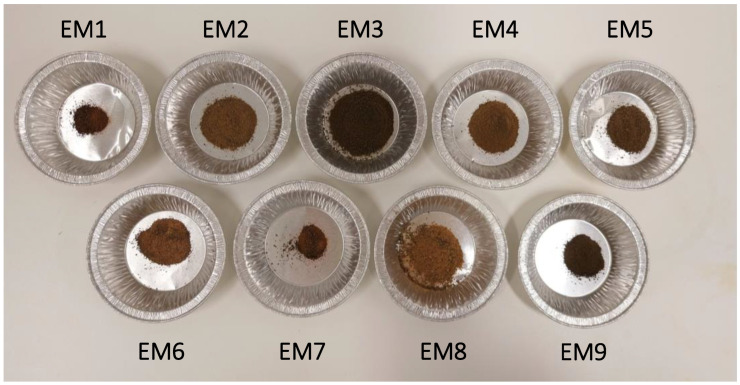
Picture of lignin samples recovered after different alkali treatments.

In a more qualitative way, the appearances of pulp and lignin samples after treatment are presented in Figure 3 and Figure 4. Harsher conditions yielded paler pulp, whereas milder treatments resulted in visible brown discoloration, likely due to residual lignin. Furthermore, pulps obtained under high temperatures and NaOH concentrations formed compact particles. One possible explanation is that extensive hemicellulose removal during alkali treatment allowed cellulose fibrils to form new hydrogen bonds, leading to closer fiber packing [22].

Alkali treatment of corncob demonstrates good efficiency with interesting lignin yields. The two reaction parameters showing the greatest effect on treatment effectiveness are NaOH concentration and temperature. A significant increase in lignin yields for higher temperature and NaOH concentrations was correlated with a drop in post-treatment pulp yield. The question of the purity of the lignins obtained must nevertheless be raised, as certain conditions also lead to the recovery of a lot of hemicellulose. This question can be answered by the NREL analysis of the samples (see Section 3.3).

Comparison with the literature confirmed that optimized alkaline pretreatment of corncob, aimed at maximizing lignin removal, enhances enzymatic hydrolysis by cellulases [23]. The optimum conditions published by the referenced authors were a NaOH concentration of 1%, a temperature of 50 °C, and a treatment time of 4 h. They found that alkaline pretreatment reduced the lignin content of corncob from 14% to 7%, while increasing the cellulose content from 36% to 50%. These processing conditions are also close to the optimum (EM4) condition of this study. The EM4 condition was carried out with a NaOH concentration of 1.5%, a temperature identical to Sahare et al. [23] of 50 °C and a time of 1 h. The authors also demonstrated that alkaline treatment of corncob changed its structural properties, increasing its surface area and decreasing its crystallinity, making it more easily hydrolysable by enzyme. This suggests the potential for dual valorization of corncob following alkaline treatment: lignin can be recovered and utilized, while the cellulose-rich pulp, rendered more accessible to cellulolytic microorganisms, can be employed in energy applications such as bioethanol or biomethane production.

### 3.2. Organosolv Treatment of Corncob

The conditions were determined in support of previous work on Brewer’s spent grain [24]. Delignification with organic solvents works by hydrolysis of α-aryl-ether and lignin–hemicellulose links [25]. So, as previously stated, pulp yield and lignin yield are relevant criterions for discriminating the best conditions.

**Figure 5:**
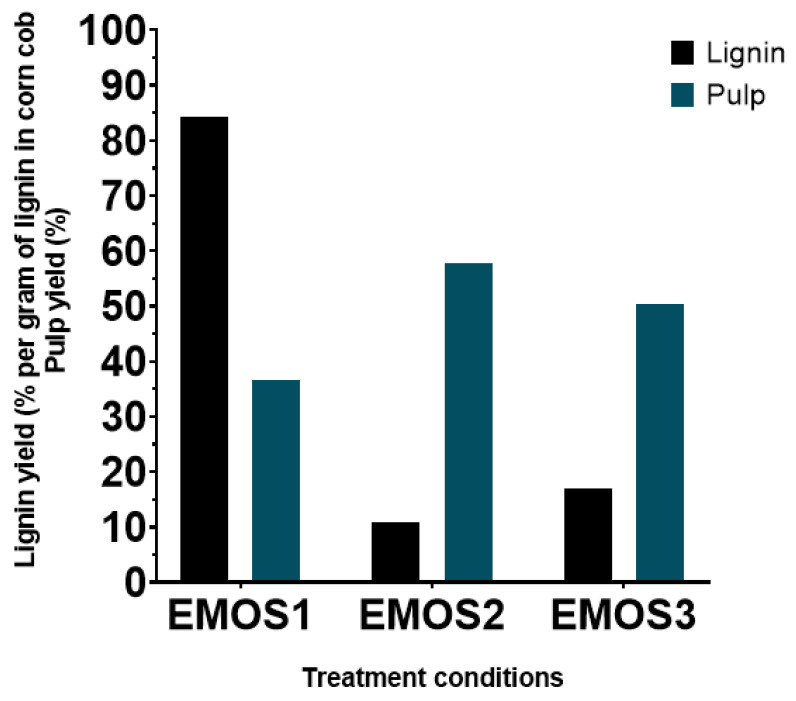
Lignin and pulp yields obtained after different organosolv treatments of corncob in PARR reactor. EMOS1 was carried out at 200 °C, EMOS2 at 160 °C, and EMOS3 at 180 °C.

Results are summarized in Figure 5. The chosen organosolv treatment conditions showed poor efficiency under 200 °C with a lignin yield of only 17% at 180 °C (EMOS3) and 11% at 160 °C (EMSO2), compared to a yield of 84% at 200 °C (EMOS1). This finding is confirmed by pulp yield with a recovery of, respectively, 58% and 51% at 180 °C and 160°C versus 37% for 200 °C. These results seem to confirm that delignification of corncob is greater at higher temperatures, making temperature a significant factor for organosolv pretreatment [26]. These differences can also be explained by the fact that reactions are carried out in the Parr reactor, a closed vessel, where another factor must be considered: pressure. Indeed, the pressures in the reactor were approximately 8 bars at 160 °C, 14 bars at 180°C, and 23 bars at 200 °C.

**Figure 6:**
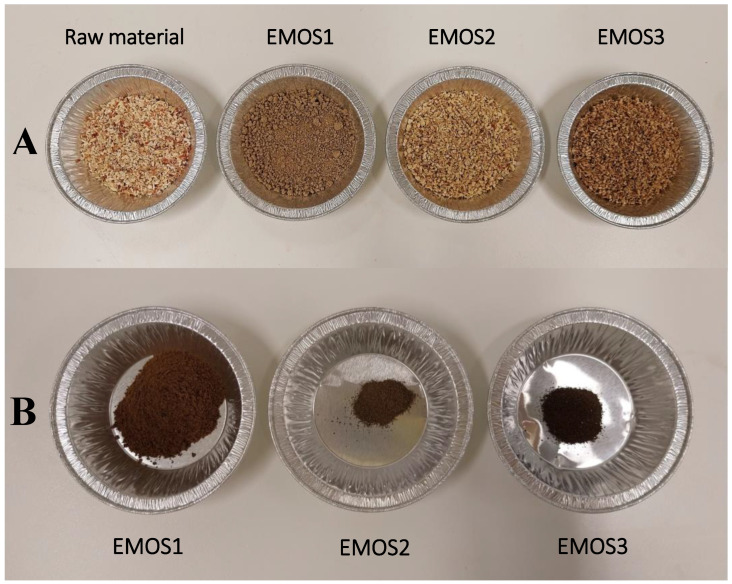
Picture of (**A**) pulp samples and (**B**) lignin samples after organosolv treatment of corncob in a Parr reactor at different temperatures.

The appearance of dried samples after treatment (Figure 6) is also a good confirmation of the previous findings. While few differences were observed in the appearance of the lignins, this was obvious for the pulp. The fibers were completely destructured in the 200 °C condition, whereas the pulp recovered from the lower-temperature treatments was very close to the starting raw material.

The pH of the black liquor at the reactor outlet, just after filtration, was 4.5. This value corresponds to the optimum pH for the activity of most laccases. This is even more interesting given that this enzyme is capable of catalytic activity in the presence of ethanol up to 37 to 40% [27]. Laccase is also capable of working in other organic solvents that can be used for lignin extraction [28]. This means that enzymatic treatment of black liquor, without the steps of precipitation, washing, centrifugation and drying, could potentially be envisaged.

Other researchers have investigated the organosolv treatment of corncobs. In the work of Buyukoztekin and Buyukkileci [29], they have also pretreated corncob in a pressure reactor by using 70% ethanol at 170 °C for 1 h. Under those conditions, approximately 50% of the lignin was successfully removed. In contrast, the study by Lv et al. [30] employed a distinctly different solvent system, a water–tetrahydrofuran (H_2_O-THF) (3:7, *v*/*v*) mixture, to effectively isolate lignin from corncob residue.

Given the ecological goal of this study of valorizing extracted lignin alongside agricultural by-products, assessing the environmental footprint of the employed extraction processes, whether alkaline or organosolv, becomes particularly important. In fact, both extraction methods have environmental drawbacks. Alkaline extraction uses caustic chemicals like NaOH, creating wastewater that requires neutralization, while organosolv methods rely on organic solvents such as ethanol, methanol, and acetone, which present challenges related to flammability, toxicity, and solvent recovery. To make these processes more sustainable, several strategies exist and focus on solvent reuse, greener reagents, and process integration to reduce waste and resource consumption. Thus, modern organosolv processes often incorporate solvent recovery systems, such as distillation or closed-loop setups, achieving solvent recovery rates over 90%, which reduces both environmental impact and costs [31,32]. Innovations also include the use of biodegradable, green solvents, such as water, food-grade solvents like coffee furanone, or deep eutectic solvents, which are less toxic and more eco-friendly. For example, the “CoffeeCat” process employs biodegradable solvents and natural amino acids to selectively extract lignin under mild conditions, minimizing environmental harm [33]. Some researchers are also exploring non-toxic reagents like amino acids or mild organic acids as substitutes for more harmful mineral acids or bases, which helps decrease hazardous waste. Additionally, alternative extraction technologies, such as supercritical fluids, microwave-assisted extraction, and ultrasonic cavitation, offer promising possibilities to reduce solvent use, energy consumption, and reliance on hazardous chemicals, contributing to a more sustainable extraction process overall [34]. Although we have stuck to conventional extraction techniques in this work, opportunities exist to make these processes more environmentally friendly, which could be fruitfully explored in subsequent research.

### 3.3. Chemical Analysis of Lignins

As already mentioned, the extracted lignins did not consist solely of lignin, but most probably also contain hemicellulose or other oligosaccharides derived from fiber degradation. To answer this question, an analysis of biomass composition was performed using the NREL procedure. This methodology uses a two-step hydrolysis to fractionate the biomass into forms that are more easily quantified. Lignin can be defined as acid-insoluble and acid-soluble lignins. Poly- and oligosaccharides are easily hydrolyzed into monosaccharides which can be identified with HPLC. The NREL procedure also considers ash and protein by gravimetric analysis [19].

**Figure 7:**
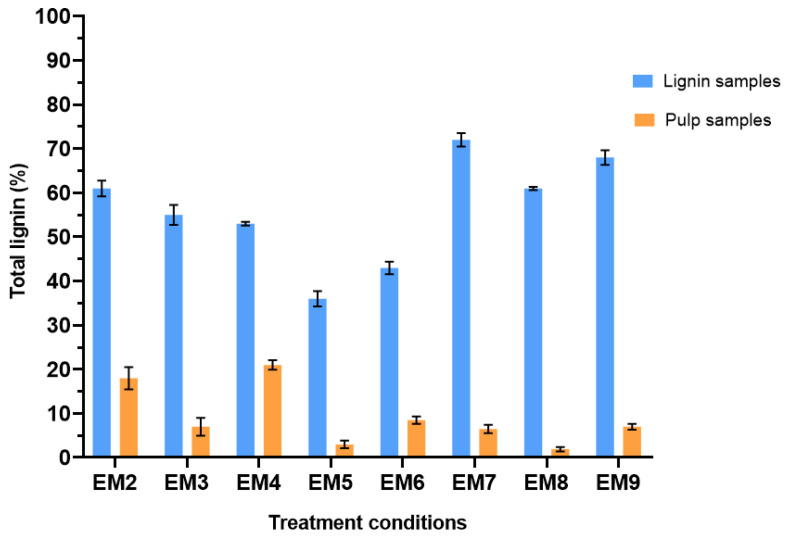
Evaluation of the real lignin content of both lignin and pulp samples collected after an alkali treatment.

**Figure 8:**
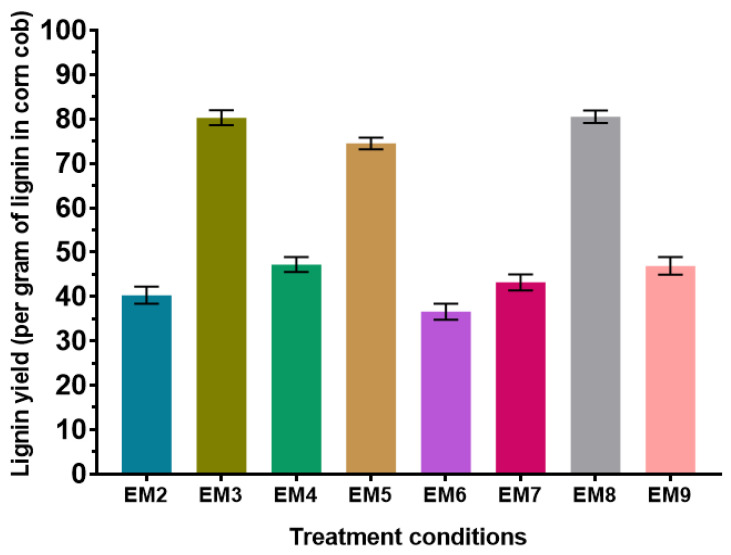
Real lignin yields obtained after alkali treatments of corncobs, considering the actual lignin content, in the powder harvested after drying.

The analysis of raw lignin and pulp samples from the alkaline treatment of corncob are summarized in Figure 7. It should be noted that condition EM1 could not be analyzed because the quantities of lignin obtained after treatment were too low, even after several replicates. From these analytical values, “real” lignin yields per treatment condition were calculated by integrating the percentage of lignin in the samples with the previously calculated yields just after treatment (Figure 2). These new lignin yields are summarized in Figure 8.

In the light of this compositional analysis, the two best yields obtained correspond to experiments EM3 and EM8, both achieving yields of 82%. These conditions are the one conducted at the higher temperature of 80 °C for 1% and 1.5% NaOH concentration. The most significant factor for the extraction of lignin from corncob by alkaline treatment is therefore temperature, with a clear increase in efficiency as temperature rises. The condition EM5 with 3% NaOH and a temperature of 50 °C is also interesting, with a final lignin yield of 74%. These results are consistent considering the lignin remaining in the pulp samples after treatment (7% for EM3, 2% for EM8, and 3% for EM5). This result proves that these conditions enable maximum recovery of the lignin present in corncobs. HPLC results on alkali-treated samples confirm the hypothesis that some lignin samples are contaminated with hemicellulose, particularly in high-temperature conditions. Concentrations of xylose, the major component of hemicellulose chains, were 0.122 g/L for condition EM3 treated at 80 °C with 1.5% NaOH. They were also high for condition EM7 treated at 50 °C with 1.5% NaOH (0.609 g/L). All lignin samples contain glucose, which can originate from both degraded cellulose and hemicellulose.

The results obtained for lignin and pulp samples from organosolv treatment were different from those expected. The pulp samples of all three organosolv conditions were analyzed by NREL analysis. The results gave, as expected, an extremely low percentage of residual lignin in the pulp after treatment. This percentage is the lowest of all the treatments carried out, alkaline and organosolv combined, as the EMOS2 and EMOS3 treatments led, respectively, to 10.8 and 10.0% of lignin in the pulp. These findings support the treatment’s efficiency and reflect the altered, destructured appearance of the pulp following treatment at 200 °C in the PARR reactor. However, the results of the analysis of the lignin recovered after the EMOS1 treatment are more surprising. The sample contained only 37.5% lignin, much lower than alkaline treatments. Given the high delignification efficiency and the minimal lignin content remaining in the treated pulp, the most plausible explanation is an inefficiency in the lignin recovery protocol post-treatment. In this case, lignin was precipitated by the addition of water. However, the supernatant obtained after centrifugation exhibited a residual brown coloration, indicating incomplete lignin precipitation and suggesting that a portion of the solubilized lignin remained in the liquid phase. Lignin precipitation from liquors generated by organosolv treatment is commonly performed through the addition of water. This approach is extensively documented in the literature, which also outlines several potential strategies for optimizing this precipitation process. The precipitation time in water can be modified to recover more lignin, for example Faruq and Nusirat [35] performed their precipitation for 1 h under constant stirring. Another approach is to modulate the volume of water added to the liqueur and the number of additions. For example, in the study by Jasiukaitytė-Grojzdek et al. [36], several protocols were tested and studied. The addition of 300 mL of water three times, 100 mL three times, and then 600 mL once or even 50 mL six times followed by 600 mL once. They were able to demonstrate that for the first protocol with 300 mL of water added three times, the larger lignin macromolecules were precipitated with the first addition of water and analogous oligomeric lignin was precipitated with the second and third addition. When the water was added more gradually for the two other protocols with smaller volumes, they also noticed that the precipitation steps start with the largest lignin macromolecules and finish with the smallest ones. Thus, enhancing the fractionation steps and reducing the volume of water per addition may improve the recovery of low-molecular-weight lignin molecules, which are likely those that were not precipitated under the chosen conditions.

To contextualize the significance of the observed lignin yields, it is crucial to compare these results for corncobs with data from other studies involving lignin-rich feedstocks. For instance, notably high lignin yields have been reported in studies involving rice straw. Lam et al. [37] demonstrated that rice straw, with an initial lignin content of 18.3%, underwent delignification when using formic acid at elevated temperature (100 °C) for 60 min. Their process resulted in lignin yields of 83.1%, very similar to those obtained for the corncob in this study, along with a minimal residual lignin content in the pulp of approximatively 3%. Other studies corroborate that the lignin yields from corncobs in this work are comparable to those reported for other grassy biomass feedstocks using optimized extraction methods. For instance, Wildschut et al. [38] achieved a lignin yield of 84% from wheat straw using the organosolv process with the same solvent (50% *w*/*w* aqueous EtOH) at a slightly higher temperature of 210 °C. Huang et al. [39] achieved a lignin yield of 89.8% by subjecting sugarcane bagasse to a treatment with alkali hydrogen peroxide.

These results highlight that corncobs, along with other feedstock by-products, can serve as promising sources of lignin with high extraction yields. This is true even though the extraction methods vary depending on the plant species and their characteristics and chemical compositions.

The lignin samples obtained by alkaline treatment were also analyzed with FTIR to see whether the differences in treatment affect its chemical structure. The assigned band wavelength is described in Table 3 [20,40].

It is important to consider that the lignin purity varies across different samples, resulting in different lignin contents (see Figure 8). This variability likely affects the appearance of the obtained IR spectra, and thus interpretations, particularly of peak intensities, should be approached with caution.

Looking at the effect of reaction time on the infrared spectra of lignin samples, comparing for example the spectra obtained for samples EM1 and EM2 (Figure S1), both recovered after treatment at 22 °C with 1.5% NaOH concentration but after 1 h and 24 h, respectively, the only real notable difference is the disappearance for EM2 of the band at 1127 cm^−1^ corresponding to the aromatic C-H and C=O stretching. The same observation is made considering the effect of NaOH concentration on the infrared spectra of lignin, all treated at 50 °C for 1 h (Figure S2). For the highest concentration of 3% NaOH (EM5), this band also disappears at 1127 cm^−1^. Qualitatively, this high concentration also shows a sharp drop in the intensity of all peaks, perhaps indicative of a harsher, slightly lignin-denaturing treatment. According to Faix et al. [41], the decrease in the 1127 cm^−1^ band is normal in the case of alkaline hydrolysis. A more detailed explanation is given by Derkacheva et al. [42], establishing that alkali treatment is responsible for the cleavage of some aryl-ether bonds, associated with an increase in phenolic hydroxyl content, that is visible on infrared spectrum.

Finally, temperature (Figure S3) appeared to be the most significant parameter in the alkaline treatment of corncob. The most noticeable difference is visible at 1650 cm^−1^, the rest of the spectrum, although different in intensity, shows bands at similar wavelengths. The band at 1650 cm^−1^, which is present only for the EM1 condition treated at 22 °C, is much less visible for treatments at 50 °C and 80 °C. According to Table 3, this band corresponds to C=O stretching in conjugated p-substituted aryl ketones.

**Figure 9:**
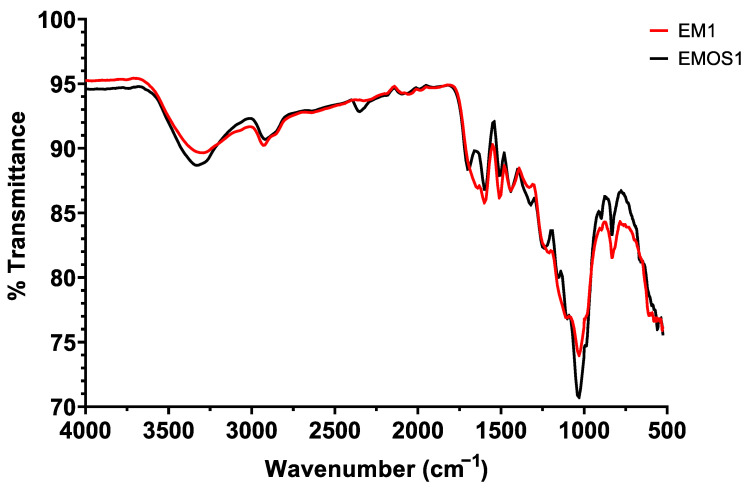
FTIR spectra of EM1 lignin samples obtained via alkaline treatment and EMOS1 lignin obtained via organosolv treatment.

The FTIR spectra of lignin obtained with an alkali treatment of corncob (EM1) and lignin obtained with an organosolv pretreatment (EMOS1) were compared (Figure 9). The main difference on the spectra is in the 1750–1600 cm^−1^ region. For alkali lignin, a band at 1650 cm^−1^, already assigned to C=O stretching in conjugated p-substituted aryl ketones, is absent on the spectrum of organosolv lignin. On the other hand, organosolv lignin has an extra band at 1701 cm^−1^ compared to alkali lignin. This band can be assigned to unconjugated ketones, carbonyls, and esters groups, conjugated aldehydes, and carboxylic acids. Organosolv lignin also has an extra band at 1240 cm^−1^ that can be attributed to a syringyl ring and C-O stretching [43].

The chemical analysis of lignins produced made it possible to determine the most effective treatment conditions for obtaining good yields with relatively pure lignin, and to understand that the temperature factor had the greatest influence on the yield and quality of the lignin produced by alkaline treatment. Finally, FTIR analysis confirmed these hypotheses and highlighted the fact that lignins produced by organosolv and alkaline treatments had slightly different chemical structures, which was expected and is probably responsible for their properties and, notably, the solubility being known to diverge.

### 3.4. Enzymatic Modifications of Lignins

Laccases are known to catalyze reactions on lignin and are then often referred to as lignolytic enzymes. Although, their catalytic mechanisms remain difficult to predict. Due to their potential for numerous industrial applications, this study aimed to explore the reactivity of a fungal laccase on different lignins extracted from corncobs.

The first observation is that the fungal laccase from *Pycnoporus cinnabarinus* exhibited no discernible catalytic effect on the organosolv lignin treated in the Parr reactor. This is evidenced by the FTIR spectra, which remained unchanged following enzymatic treatment with just a slight difference in intensity (Figure S4). This result was anticipated, given that organosolv lignins exhibit poor solubility in water and limited solubility under acidic conditions, the optimal pH range for most fungal laccases. A study by Milstein et al. [44] demonstrated that under such conditions, the lignin underwent significant transformation, including an increase in phenolic groups, alterations in the number of conjugated structures, and changes to both the aliphatic and aromatic carbon components of the lignin molecule.

**Figure 10:**
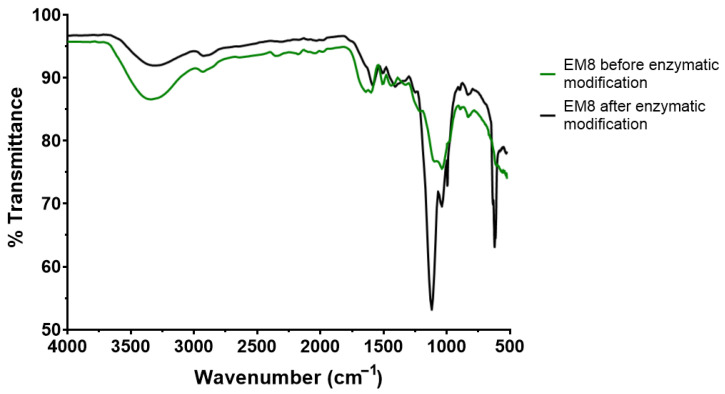
Example of an FTIR spectra with alkaline lignin EM8 before and after laccase treatment.

In contrast, lignin isolated thanks to alkaline treatment of corncobs showed more promising results as they showed significant modifications by laccase (Figure 10). All FTIR spectra of all laccase-treated alkaline lignins show a decrease in intensity in the 3500–3100 cm^−1^ spectral region. This band is characteristic of the stretching vibrations of hydroxyl groups including phenolic hydroxyl groups and hydrogen bonding. The reduction in intensity in this region suggests oxidation of phenolic groups by laccase, which is consistent with the enzyme’s known mechanism [45]. A significant decrease in intensity and even disappearance is also observed in the 1700–1650 cm^−1^ region for modified lignin. As this spectral region is associated with C=O stretching in unconjugated ketones, carbonyls, and ester groups or conjugated aldehydes and carboxylic acid, this suggests a change or elimination of some carbonyl groups. This observation could be the result of radical coupling, decarboxylation, or condensation reaction of the lignin. Notable changes are also visible in the 1300–1000 cm^−1^ region, which contains bands associated with C-O stretching in ester or ether groups and aromatic C-H vibrations. Particularly noteworthy are changes in the bands at 1130 cm^−1^ and 1105 cm^−1^. These modifications may indicate changes in ether bonds (β-O-4) and structural rearrangements affecting C-O bonds. Some of these changes could result from the breaking of ether bonds or the formation of new ones. Observations like those observed here are listed in the study by Zhang et al. [46] which was also carried out on lignin from corncobs. All these observations point to lignin degradation and depolymerization by *Pycnoporus cinnabarinus* laccase.

DSC analyses were also conducted on both the native lignin samples and the lignins treated enzymatically. The parameter studied here was the glass-transition temperature, (T_g_), which marks the reversible change in free volume and molecular mobility of the amorphous lignin network, delineating the glassy and rubbery domains. In polymers, T_g_ is intimately related to chain length and cross-link density via the Fox–Flory equation [47]. A qualitative decrease in T_g_ values therefore suggests a reduction in molecular weight: shorter polymer chains exhibit higher mobility and can act as plasticizers, reducing the energy required to reach the glass-transition point, which subsequently occurs at lower temperatures.

One notable observation is the difference in T_g_ between the two types of lignin extracted from corncobs. Organosolv EMOS1 lignin exhibits a T_g_ of 161 °C, whereas all lignins obtained through alkaline treatment show significantly higher T_g_ values, ranging from 209 °C to 222 °C, depending on the specific treatment conditions. We can therefore see quite clearly that lignin obtained by organosolv treatment has a lower molecular weight, with shorter chains, than alkaline lignins. Once again, this result is consistent with the literature that usually reports organosolv lignin in the range 0.5 to 10.8 kDa, compared to 1.5 to 5.0 kDa for kraft lignin and 5.0 to 50.0 kDa for lignosulfonates [48]. This is also consistent with earlier findings on lignin recovery through water precipitation during organosolv treatment, indicating that we did not primarily recover low-molecular-weight lignin that probably tended to remain in the supernatant. The particularly high T_g_ values observed for the alkaline lignins exceed those commonly reported in the literature, typically between 120 °C and 180 °C, suggesting that additional mechanisms are at play [49]. In addition to extensive condensation reactions under hot alkaline conditions, which promote the formation of rigid C–C inter-unit linkages and reduce chain mobility, rapid repolymerization of lignin fragments appears to contribute significantly. During alkaline extraction, cleavage of β-O-4 linkages generates phenoxy radicals that can undergo fast radical coupling, resulting in new aromatic C–C and benzyl ether bonds. This process actively increases effective molecular weight and cross-link density in situ, supporting the unusually high T_g_ values.

This dual mechanism is further corroborated by FTIR analyses: the disappearance of the β-O-4 ether band at 1127 cm^−1^ (indicative of extensive cleavage), as well as the emergence and intensification of aromatic C–C stretching near 1595–1605 cm^−1^, both point to the formation of new condensed structures via radical-mediated repolymerization. Such spectral changes, together with the observed increase in DSC endothermic enthalpy, reflect the development of a rigid, cross-linked network that characterizes these alkaline lignins.

These findings suggest that the rapid repolymerization of lignin fragments, kinetically favored under the high-temperature and alkaline environment, complements classical condensation, together accounting for the observed T_g_ elevation, which can reach up to 60 °C above that of organosolv lignin. As a result, the glass-transition behavior of the alkaline lignins cannot be solely attributed to molecular weight effects but must also consider the impact of network architecture generated during extraction.

For alkaline lignins, a decrease in T_g_, and therefore molecular weight, is also observed after enzymatic treatment with laccase for conditions EM4, EM5, EM7, and EM8 but not for conditions EM6 and EM3. This reduction is attributed to the radical-mediated cleavage of residual β-O-4 bonds by the laccase, generating shorter, more mobile fragments and increasing free volume, which counteracts the effects of condensation and repolymerization. In contrast, organosolv lignin exhibits negligible changes in T_g_ after enzymatic treatment, likely due to its poor dispersibility and the limited accessibility of the enzyme under the conditions employed.

## 4. Conclusions

This work highlights the successful implementation of alkaline and organosolv pretreatment strategies for the extraction of lignin from corncob, a largely undervalued agricultural by-product. Both methodologies offered high lignin recovery efficiencies, with optimal yields of 82% and 84% for alkaline and organosolv protocols, respectively, and clearly demonstrated that temperature is a critical determinant for maximizing extraction performance. Through comprehensive spectroscopic analysis, this study established that each extraction route yields lignins with distinct structural features, underscoring their non-equivalence in terms of subsequent chemical reactivity and potential applications.

Notably, only alkaline lignin underwent significant structural modifications upon enzymatic treatment with *Pycnoporus cinnabarinus* laccase, reflecting its higher aqueous compatibility and more accessible structure under the chosen reaction conditions. The lack of observable modification in organosolv lignin is most plausibly attributed to its limited solubility and higher hydrophobicity at the acidic pH optimal for fungal laccases. These findings not only advance the mechanistic understanding of extraction-dependent lignin reactivity but also support strategic selection of process routes according to desired downstream modification or valorization.

The broader significance of this study lies in its contribution to the comprehensive valorization of corncob biomass. By enabling selective extraction and enzymatic tailoring of lignin fractions, it supports the development of sustainable biorefinery concepts aimed at minimizing waste and maximizing value generation from agricultural residues. Furthermore, the results demonstrate that the fractionated lignins obtained hold promise for high-value applications, such as specialty polymers, green adhesives, or functional materials. Future research should address scale-up feasibility. Indeed, biomass variability poses significant challenges in lignin extraction processes, as agricultural residues like corncobs differ naturally in chemical composition, moisture content, and particle size, all of which can influence extraction efficiency and product quality. Moisture content and particle size variability affect solvent penetration and reaction kinetics, making process outcomes less predictable if not carefully controlled. To achieve consistent and repeatable results across different biomass batches, strict process standardization, including standardized pretreatment steps, controlled operating conditions, and real-time monitoring, is essential [50].

Finally, the prospects for engineering more effective enzymatic systems, including laccases with broader pH and solvent compatibility or combined mediator-assisted protocols, warrant exploration to unlock the full reactivity spectrum of structurally diverse lignins. Overall, these advances align closely with the principles of the circular bioeconomy and contribute to the transition toward more sustainable material systems.

## Figures and Tables

**Table 1:** Different experimental conditions tested for the optimization of the alkali treatment of corncob.

Samples	Conditions			
	NaOH (%)	Temperature (°C)	Time (h)	Solid–Liquid Ratio
EM7	1	50	1	1:20
EM8	1	80	1	1:20
EM9	1	22	24	1:20
EM1	1.5	22	1	1:20
EM2	1.5	22	24	1:20
EM3	1.5	80	1	1:20
EM4	1.5	50	1	1:20
EM5	3	50	1	1:20
EM6	3	22	1	1:20

**Table 2:** Different experimental conditions tested for the optimization of the organosolv treatment of corncob.

Samples	Conditions			
	Solvent	Temperature (°C)	Time (h)	Solid-Liquid Ratio
EMOS1	50% aq.EtOH, pH 3.5	200	0.5	1:20
EMOS2	50% aq.EtOH, pH 3.5	160	0.5	1:20
EMOS3	50% aq.EtOH, pH 3.5	180	0.5	1:20

**Table 3:** Main FTIR absorption bands of lignin samples.

Band Wavelength (cm^−1^)	Vibration Band Assignment
3600–3200	OH groups and hydrogen bonds
2930	Methyl groups, methoxyl C-H stretching
1700	Unconjugated C=O stretching, conjugated aldehydes and carboxylic acid
1600	Conjugated C=O streching
1511	C=C aromatic skeletal stretching
1452	C−H deformation asymmetric in CH_2_ and CH_3_, with C−H in-plane deformation
1420	Aromatic skeletal with C-H in-plane deformation
1321	S-ring ring breathing with C=O stretching, or G-ring substituted in C5
1239	C-C, C-O and C=O stretching of G unit
1157	C=O stretching in conjugated ester
1121	Aromatic C-H deformation, C=O stretching
1105	Aromatic C–H in plane deformation in S-unit
1030	Aromatic C-H in plane deformation, C-O deformation in primary alcohols, C=O stretching (unconjugated)
831	C-H of G units

## Data Availability

Data is contained within the article or Supplementary Material; Dataset available on request from the authors.

## Supplementary Materials

The following supporting information can be downloaded at: https://www.mdpi.com/article/10.3390/biom15091226/s1. Figure S1: FTIR spectra of lignins EM1 and EM2, effect of reaction time; Figure S2: FTIR spectra of lignin samples EM4, EM5 and EM7, effect of NaOH concentration; Figure S3: FTIR spectra of lignin samples EM1, EM3 and EM4, effect of temperature; Figure S4: FTIR spectra of lignin samples EMOS1, before and after enzymatic treatment.
