# Supplementary material for: From Corncob By-Product to Functional Lignins: Comparative Analysis of Alkaline and Organosolv Extraction Followed by Laccase Treatment
Source: Biomolecules. 2025 Aug 26;15(9):1226. doi: 10.3390/biom15091226 (PMC12467641; doi:10.3390/biom15091226)
Supplement: Supplementary file 1 [file biomolecules-15-01226-s001.zip › biomolecules-3792512-supplementary.pdf]

biomolecules

*Article*

# From Corncob By-Product to Functional Lignins: Comparative Analysis of Alkaline and Organosolv Extraction Followed by Laccase Treatment

**Elise Martin [1], Swarnima Agnihotri [2], Fabrice Audonnet [1,*], Eric Record [3], Pascal Dubessay [1], Mohammad J. Taherzadeh [2] and Philippe Michaud [1]**

[1] Clermont Auvergne INP, CNRS, Institut Pascal, Université Clermont Auvergne, 63000 Clermont-Ferrand, France; elise.martin@uca.fr (E.M.); pascal.dubessay@uca.fr (P.D.); philippe.michaud@uca.fr (P.M.)
[2] Swedish Centre for Resource Recovery, Faculty of Textiles, Engineering and Business, University of Borås, 503 32 Borås, Sweden; swarnima.agnihotri@hb.se (S.A.); mohammad.taherzadeh@hb.se (M.J.T.)
[3] INRAE, Aix-Marseille Université, UMR1163 Biodiversité et Biotechnologie Fongiques, 13288 Marseille, France; eric.record@inrae.fr
* Correspondence: fabrice.audonnet@uca.fr

## Supplementary Materials

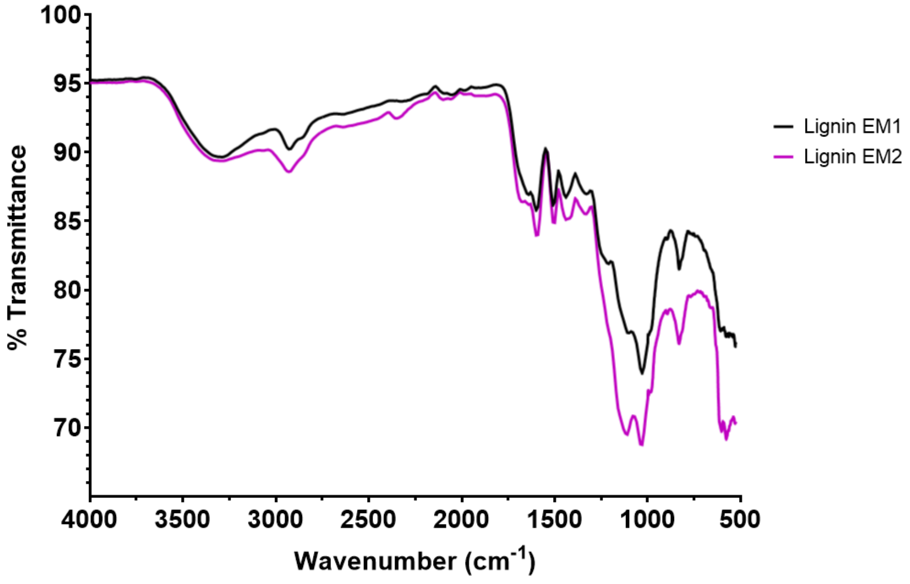


**Figure S1.** FTIR spectra of lignins EM1 and EM2, effect of reaction time.

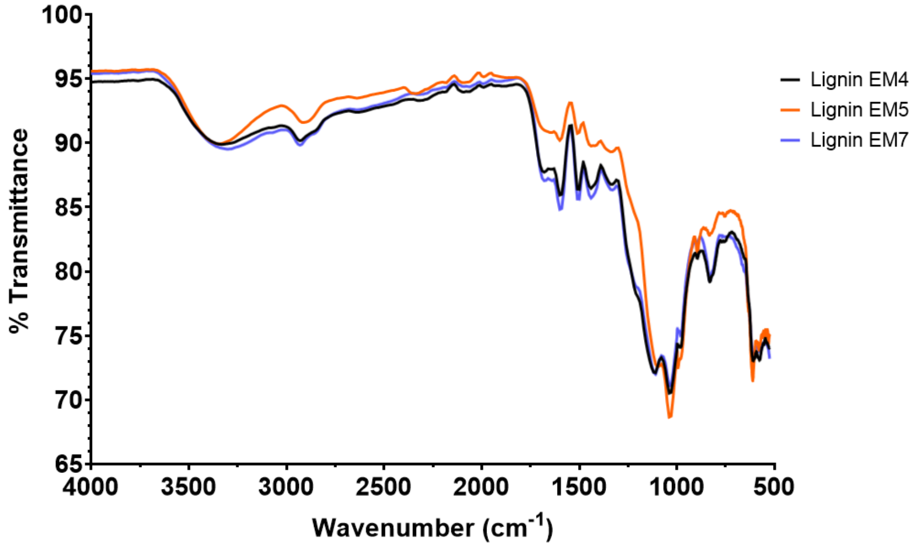


**Figure S2.** FTIR spectra of lignin samples EM4, EM5 and EM7, effect of NaOH concentration.

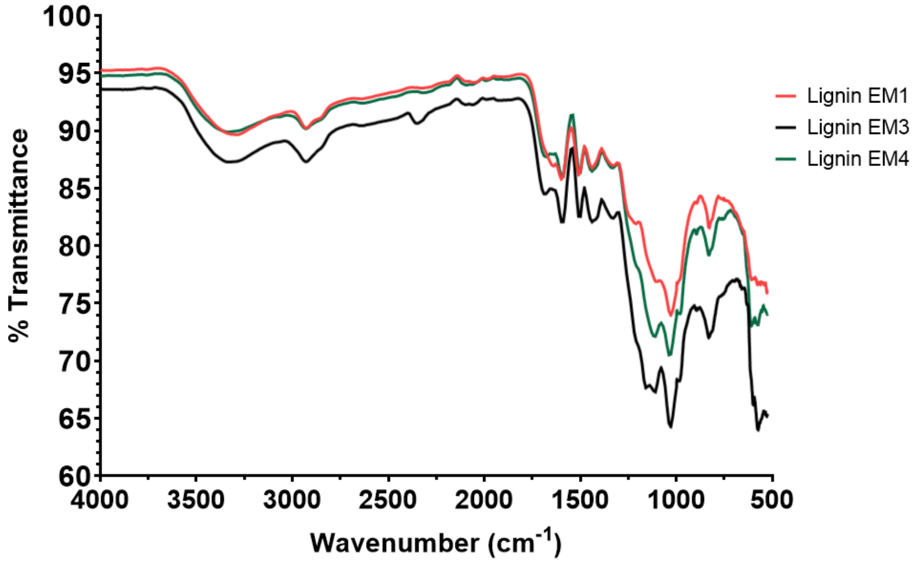


**Figure S3.** FTIR spectra of lignin samples EM1, EM3 and EM4, effect of temperature.

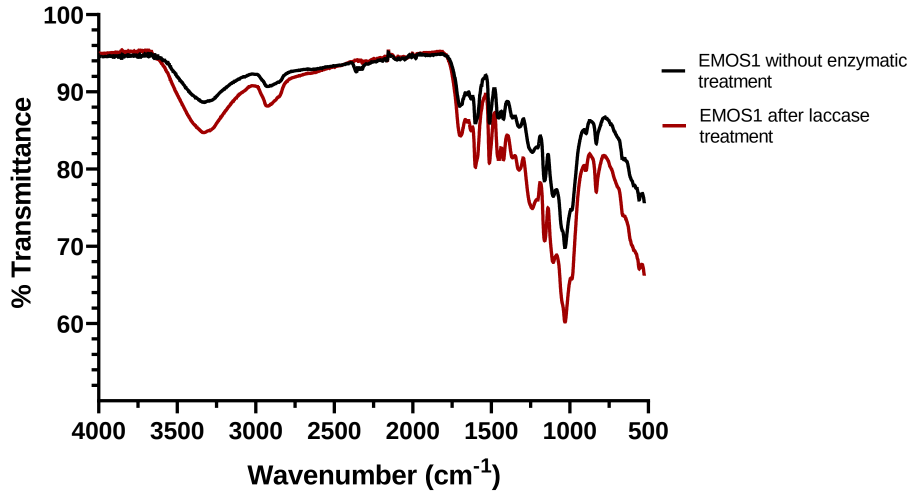


**Figure S4.** FTIR spectra of lignin samples EMOS1, before and after enzymatic treatment.
